# Tea Cultivar Genotype Shapes Rhizosphere Microbiome Assembly Through Metabolic Differentiation

**DOI:** 10.3390/plants15030414

**Published:** 2026-01-29

**Authors:** Lingfei Ji, Xiwen Fang, Shengxian Chen, Zeyi Ai, Kang Ni, Yiyang Yang, Jianyun Ruan

**Affiliations:** 1 Tea Research Center, China-Uganda Joint Laboratory for Sustainable and High-Efficiency Premium Tea Production, Jiangsu Academy of Agricultural Sciences, Nanjing 210014, China; lingfeiji@jaas.ac.cn (L.J.); shengxianchen@jaas.ac.cn (S.C.); aizeyi@jaas.ac.cn (Z.A.); 2 Institute of Leisure Agriculture, Jiangsu Academy of Agricultural Sciences, Nanjing 210014, China; 3 Key Laboratory of Biology, Genetics and Breeding of Special Economic Animals and Plants, Ministry of Agriculture and Rural Affairs, Tea Research Institute, Chinese Academy of Agricultural Sciences, Hangzhou 310008, China; nikang@caas.cn

**Keywords:** *Camellia sinensis*, microbiome structure, rhizosphere metabolites, assembly process, plant–microbe interactions

## Abstract

Tea cultivar genotype plays a critical role in shaping rhizosphere microbiome assembly, yet the underlying mechanisms remain poorly understood. This study employed a controlled pot experiment with five widely cultivated Chinese tea cultivars (*Camellia sinensis*) to investigate how cultivar-specific variation influences rhizosphere microbial communities and their assembly processes. Rhizosphere soil microbiomes (bacterial and fungal communities) and metabolomes were characterized using 16S rRNA and ITS2 amplicon sequencing combined with untargeted metabolomics. Significant differences in rhizosphere metabolite composition, primarily organic acids, fatty acids, and carbohydrates, were observed among cultivars, which corresponded to distinct bacterial and fungal community structures. Redundancy analysis (RDA) revealed that rhizosphere metabolites explained 19.87% of bacterial and 21.63% of fungal community compositional variation, second only to soil physicochemical properties. Neutral community model and modified stochasticity ratio analyses indicated that microbial assembly across cultivars was predominantly deterministic, and rhizosphere metabolite profiles were strongly correlated with microbial community structure. Notably, arbuscular mycorrhizal fungi made up about 11% of the fungal communities in minimally fertilized pot systems, contrasting sharply with their near-absence in conventionally managed systems plantations. These findings demonstrate that tea cultivar genotype significantly shapes rhizosphere microbiome assembly through metabolic differentiation, providing a theoretical foundation for integrating microbiome considerations into tea breeding programs and developing cultivar-specific management strategies.

## 1. Introduction

Plant–rhizosphere microbiome interactions have emerged as a pivotal research frontier in contemporary plant biology and microbial ecology. It is increasingly recognized that plants and their associated microbiomes function as a holobiont. In this co-evolved functional unit, the host plant actively selects specific microbial communities within its rhizosphere. These recruited microbes are not merely passengers but are integral to the plant’s survival, modulating essential physiological processes such as nutrient acquisition (e.g., nitrogen fixation, phosphate solubilization), resistance to biotic stressors (pathogens and pests), and adaptation to abiotic constraints [1,2,3]. Consequently, elucidating the mechanisms governing this host-mediated selection is of significant agricultural importance. Understanding these assembly rules is a prerequisite for sustainable agriculture, underpinning the rational design of microbial fertilizers and biocontrol agents to replace chemical inputs [4]. Recent advancements in multi-omics approaches have facilitated the comprehensive identification of rhizosphere microorganisms, enabling researchers to pinpoint key microbial taxa and functional genes selected by host plants [1,3]. These insights provide a robust theoretical foundation for manipulating plant–microbe interactions to enhance crop productivity and resilience.

China exhibits an exceptionally diverse range of germplasm resources for *C. sinensis*, encompassing numerous cultivars adapted to various regional environments [5,6]. Given that tea quality and yield are inextricably linked to soil health [7], investigating how the rhizosphere microbiome responds to specific tea cultivars is crucial. Such research could provide the theoretical basis for rhizosphere-based breeding or formulating agronomic management strategies tailored to specific varieties. However, a significant gap remains in the current literature. Prior research has predominantly focused on how external factors, such as fertilization regimes, tillage, and climate, impact soil microorganisms, often limiting investigations to bulk soil or single varieties [7,8,9,10]. This external focus obscures the intrinsic role of the host genotype. Consequently, the extent to which different tea cultivars genetically impose selection pressures on their rhizosphere microbiomes, and the mechanisms driving these community assembly processes, remain largely underexplored.

To understand these assembly processes, it is essential to distinguish between root exudates and rhizosphere metabolites, though both play critical roles in shaping microbial communities. Root exudates are complex organic compounds actively released by living roots, including sugars, organic acids, amino acids, and secondary metabolites [11,12,13]. These compounds function not only as carbon and energy sources for microbial proliferation but also as sophisticated signaling molecules that mediate communication between plants and their associated microbiome [14]. In contrast, rhizosphere metabolites represent a broader metabolic pool that encompasses root exudates plus microbial-derived compounds and their transformation products, reflecting the integrated metabolic activity of the plant-microbe holobiont [13,15]. While root exudates initiate the chemical dialogue between plants and soil microorganisms, the accumulated rhizosphere metabolites represent the outcome of complex plant–microbe interactions. These metabolites collectively dictate the structure and function of the recruited microbiome, acting as filters that enrich beneficial taxa while repelling pathogens [15]. Conversely, the established microbiome can modulate metabolic pathways of the host, further altering the composition of rhizosphere metabolites [13]. Thus, the rhizosphere represents a self-reinforcing feedback loop driven by metabolic exchange.

The study of rhizosphere interactions in tea plants presents unique challenges and opportunities compared to annual crops. As a perennial woody plant, *C. sinensis* maintains a long-lived root system with distinct physiological periodicity, typically exhibiting 3–4 active growth flushes annually. The metabolic profile of tea roots is distinct, characterized by high levels of secondary metabolites, including polyphenols, alkaloids, and amino acids. For instance, recent research has demonstrated that theanine, a non-protein amino acid unique to tea, significantly alters the rhizosphere microbial community structure and selectively shapes microbial assembly [16]. Furthermore, root exudation profiles are known to evolve with increasing plantation age and developmental stages. Despite this, it remains unknown whether the genetic diversity among different tea cultivars translates into distinct rhizosphere metabolic fingerprints and whether these metabolic variations are the deterministic forces driving cultivar-dependent microbiome assembly.

To address these knowledge gaps, this study employed a controlled pot experiment using five tea cultivars widely cultivated in China. By using a controlled environment, we aimed to minimize environmental heterogeneity and isolate the specific effects of host genotype on the rhizosphere. We tested three specific hypotheses: (1) different tea cultivars produce distinct rhizosphere metabolite profiles that can be quantitatively distinguished; (2) cultivar-specific rhizosphere metabolite compositions significantly explain variations in bacterial and fungal community structures; and (3) deterministic processes driven by these metabolic differences, rather than stochastic processes, govern rhizosphere microbiome assembly across cultivars. By integrating 16S rRNA/ITS2 amplicon sequencing with untargeted metabolomics, we quantified the relative contributions of rhizosphere metabolites, soil physicochemical properties, and plant physiological factors to microbial community variation, thereby providing empirical evidence for metabolite-mediated microbiome assembly in tea plants.

## 2. Results

### 2.1. Rhizosphere Soil Physicochemical Properties, Metabolism, and Plant-Related Properties Vary Among Different Tea Cultivars

In the present study, under identical water and fertilizer management conditions, significant differences in rhizosphere soil physicochemical properties were observed among different tea cultivars (Table 1). HJY exhibited the highest pH value, which was significantly higher than those of other cultivars, whereas FDDB showed a significantly lower pH compared to all other cultivars. Additionally, HJY displayed the lowest concentrations of NH_4_^+^-N and NO_3_^−^-N in its rhizosphere among all tested cultivars. Regarding available phosphorus (AP) and available potassium (AK) contents, ZJ demonstrated the highest values for both nutrients, while TGY and HJY exhibited the lowest AK and AP contents, respectively. Furthermore, the rhizosphere soil water content of FDDB was significantly lower than that of the other cultivars. For the plant-related properties, FDDB exhibited significantly higher root biomass compared to other cultivars, whereas AJBC, TGY, and ZJ showed no significant differences in root weight among themselves. In terms of total biomass, FDDB displayed the highest value while HJY had the lowest, with this difference being highly significant (*p* < 0.05). In contrast, the three cultivars AJBC, TGY, and ZJ exhibited no significant differences in total biomass (*p* > 0.05). The root carbon-to-nitrogen (C:N) ratio showed no significant variation among tea cultivars, ranging from 50 to 60.

For the rhizosphere soil metabolism, a total of 87 rhizosphere metabolites with MS2 scores > 0.9 were detected across the five tea cultivars. The PLS-DA revealed significant differences in rhizosphere metabolite composition among different tea cultivars. The first and second axes of the PLS-DA explained 25.90% and 17.90% of the variation in metabolite composition, respectively (Figure S1). FDDB and HJY showed clear separation in their metabolite compositions, whereas AJBC, TGY, and ZJ exhibited less distinct differentiation in the PLS-DA ordination (Figure S1). VIP scores identified 41 metabolites that contributed substantially to the overall differences in rhizosphere metabolomes (Table S1). Among these, palmitic acid, neotrehalose, beta-citraurol, and 5-HETE (a hydroxyeicosatetraenoic acid) showed relatively higher abundances compared to other metabolites, with neotrehalose exhibiting significantly higher concentrations than all other detected metabolites.

### 2.2. The Microbial Alpha Diversity and Community Changes Among Different Tea Cultivars

**Figure 1:**
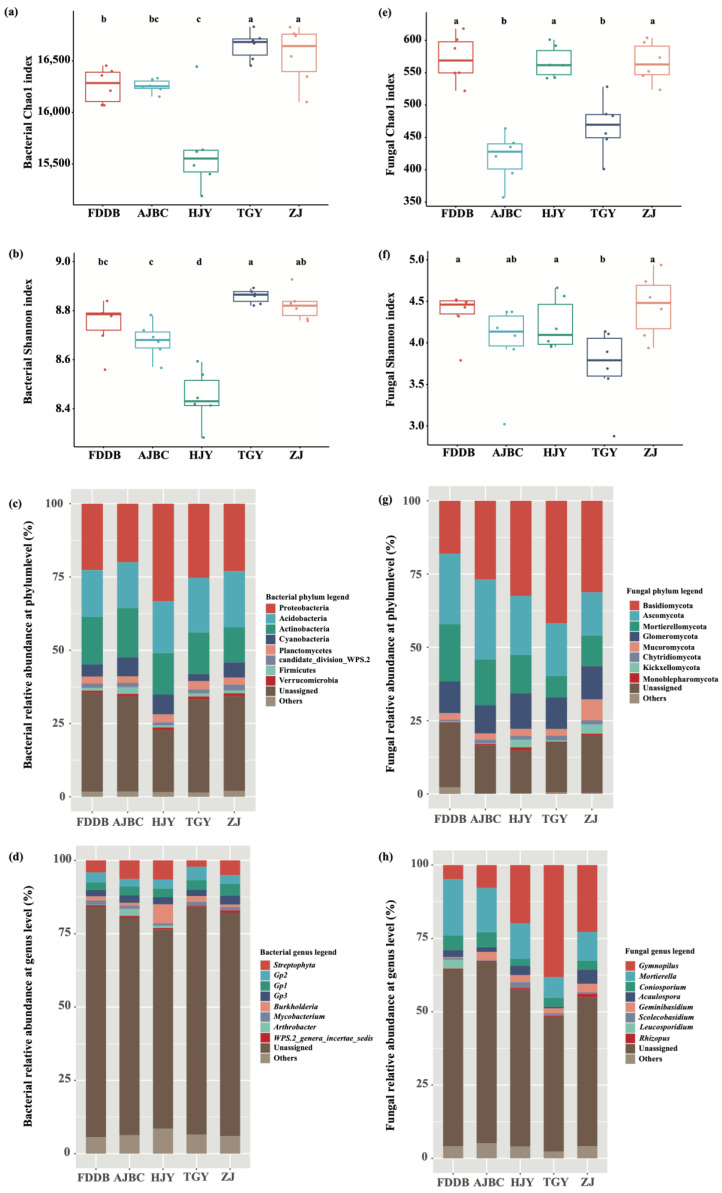
Rhizosphere microbial alpha diversity and community composition change among different tea cultivars. (**a**,**b**) show the Chao1 index and Shannon index of bacteria respectively; (**e**,**f**) show the Chao1 index and Shannon index of fungi, respectively. The different letters in each figure indicate significant differences between the treatments, *p* < 0.05. (**c**,**d**,**g**,**h**) are the relative abundance of bacterial and fungal community composition at phylum and genus level, respectively.

The Chao1 and Shannon indices of bacterial communities in the rhizosphere soil of different tea cultivars exhibited similar trends, following the order TGY > ZJ > FDDB > AJBC > HJY. Both indices were significantly lower in the HJY rhizosphere than in other cultivars (*p* < 0.05). No significant difference in bacterial alpha diversity was observed between FDDB and AJBC, though both showed relatively lower values than TGY and ZJ (Figure 1a,b). In contrast, fungal alpha diversity in the rhizosphere soil of different tea cultivars did not show patterns similar to those of bacteria. The Chao1 index of rhizosphere fungi was significantly higher in FDDB, HJY, and ZJ compared to AJBC and TGY (*p* < 0.05), while the Shannon index of fungi in TGY rhizosphere was significantly lower than in other cultivars (*p* < 0.05) (Figure 1e,f). The contrasting patterns between bacterial and fungal alpha diversity across cultivars suggest kingdom-specific responses to cultivar-mediated environmental filters. The higher fungal richness in FDDB, HJY, and ZJ rhizospheres may reflect enhanced niche partitioning among fungal guilds under cultivar-specific resource availability, while TGY’s lower fungal evenness (Shannon index) indicates dominance by specific fungal taxa (e.g., *Gymnopilus*, ~38%; Figure 1h), possibly driven by selective metabolite exudation favoring saprotrophic specialists.

The bacterial communities in the rhizosphere soil of different tea cultivars were predominantly composed of Proteobacteria (~25%), Acidobacteria (~18%), Actinobacteria (~15%), Cyanobacteria (~5%), Planctomycetes (~2%), candidate_division_WPS.2 (~2%), Firmicutes (~1%), and Verrucomicrobia (~1%) (Figure 1c). Furthermore, the relative abundance of bacteria at the phylum level varied notably across tea cultivars. For instance, the relative abundance of Proteobacteria was highest in HJY rhizosphere (~33%) and was significantly higher than in other cultivars (*p* < 0.05), while Acidobacteria and Actinobacteria reached their highest relative abundances in ZJ (~19%) and AJBC (~17%), respectively (Figure 1c). At the genus level, the dominant bacterial genera across different tea cultivar rhizospheres included *Streptophyta*, *Burkholderia*, and *Gp2*, *Gp1*, and *Gp3* from Acidobacteria, with *Burkholderia* being enriched in HJY rhizosphere at significantly higher levels than in other cultivars (*p* < 0.05) (Figure 1d).

The fungal communities in the rhizosphere of different tea cultivars were mainly composed of Ascomycota (~21%), Basidiomycota (~30%), Mortierellomycota (~13%), and Glomeromycota (~11%) (Figure 1g). The relative abundance of Ascomycota was highest in AJBC rhizosphere (~27%), significantly exceeding that in TGY (~18%) and ZJ (~15%) (*p* < 0.05). Basidiomycota showed the highest relative abundance in TGY rhizosphere, exceeding 40%, which was significantly higher than in other cultivars (*p* < 0.05). Mortierellomycota exhibited relatively high abundance in FDDB (~19%) and AJBC (~16%) rhizospheres, while being lowest in TGY (~7%). In contrast, Glomeromycota remained relatively stable across all tea cultivar rhizospheres, maintaining approximately 11% relative abundance (Figure 1g). At the genus level, the top ten genera across the five different tea cultivars were *Gymnopilus*, *Mortierella*, *Coniosporium*, *Acaulospora*, *Geminibasidium*, *Scolecobasidium*, *Leucosporidium*, and *Rhizopus*. Among these, *Mortierella* dominated in FDDB (~19%) and AJBC (~15%) rhizospheres, while *Gymnopilus* was the dominant genus in HJY (~20%), TGY (~38%), and ZJ (~23%) rhizospheres (Figure 1h).

**Figure 2:**
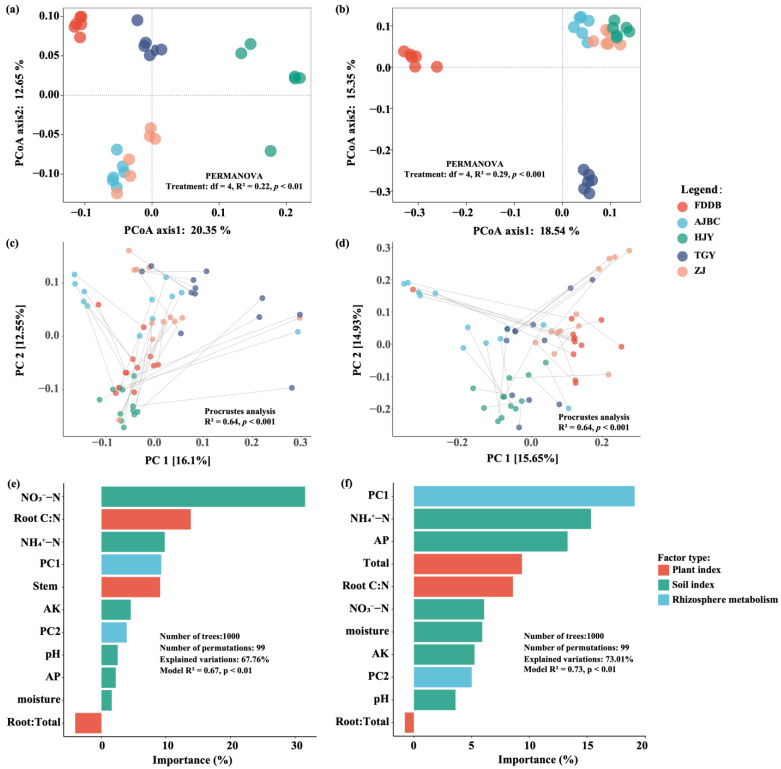
PCoA of rhizosphere soil bacterial (**a**) and fungal (**b**) communities among different tea cultivars, and PA between rhizosphere metabolome and bacterial (**c**) and fungal (**d**) microbial communities. (**e**,**f**) are the Random forest model for predicting the contributions of soil properties and climate factors for bacterial and fungal community differences, respectively.

PCoA analysis revealed significant differences in bacterial (PERMANOVA: df = 4, R^2^ = 0.22, *p* < 0.01) and fungal (PERMANOVA: df = 4, R^2^ = 0.29, *p* < 0.001) community structure among rhizosphere soils of different tea cultivars. The first two PCoA axes collectively explained approximately 33% and 34% of the variation in bacterial and fungal community structure (Figure 2a,b), respectively. Additionally, Procrustes analysis demonstrated significant associations between the rhizosphere metabolome and both bacterial communities (R^2^ = 0.64, *p* < 0.001) and fungal communities (R^2^ = 0.64, *p* < 0.001) (Figure 2c,d). RDA results indicated that soil physicochemical properties, tea plant physiological factors, and rhizosphere metabolites collectively explained 64.75% and 70.82% of the variation in bacterial and fungal community structures, respectively (Table 2). Among these factors, rhizosphere soil physicochemical properties accounted for the largest proportion of variation in both bacterial (23.40%) and fungal (24.93%) community structures. Rhizosphere metabolites explained 19.87% of bacterial community variation and 21.63% of fungal community variation, while tea plant physiological factors contributed the least to community structure variation, explaining 13.41% and 11.88% for bacterial and fungal communities, respectively. Random Forest modeling further revealed that NO_3_^−^-N and root carbon-to-nitrogen ratio were the two factors contributing most to bacterial community variation across different tea cultivar rhizospheres, with the model explaining 67.76% of bacterial community structure variation (Model R^2^ = 0.67, *p* < 0.01). For fungal communities, rhizosphere metabolite composition and NH_4_^+^-N were identified as the most important factors driving community variation among different tea cultivars, with the model explaining 73.01% of fungal community structure variation (Model R^2^ = 0.73, *p* < 0.01) (Figure 2e,f).

### 2.3. The Assembly Process and Function Fluctuation of Rhizosphere Soil Microbial Communities Among Different Tea Cultivars

**Figure 3:**
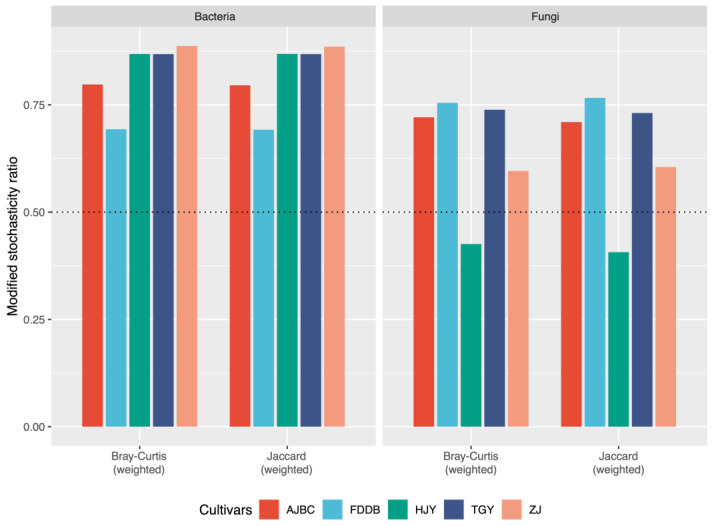
Modified stochasticity ratio of rhizosphere bacterial and fungal communities based on Bray–Curtis distance and Jaccard distance in different tea cultivars. The dotted line in the figure represents the threshold of 0.5.

MST analysis revealed that deterministic processes predominantly governed the assembly of bacterial communities in the rhizosphere of different tea cultivars, with MST values based on Bray–Curtis distance all exceeding 0.5 (Figure 3). Similarly, deterministic processes also dominated the assembly of fungal communities in the rhizosphere of different tea cultivars, with the exception of HJY (Figure 3). The MST values based on Jaccard distance exhibited trends consistent with those based on Bray–Curtis distance. The NCM results further demonstrated that stochastic processes did not play a dominant role in the assembly of bacterial communities across different tea cultivar rhizospheres, as evidenced by the low model fit of NCM for each cultivar (R^2^: 0.14–0.25). Similar patterns were observed for fungal community assembly across different tea cultivars, with the NCM model fit also remaining low (R^2^: 0.04–0.27) (Figure S2).

**Figure 4:**
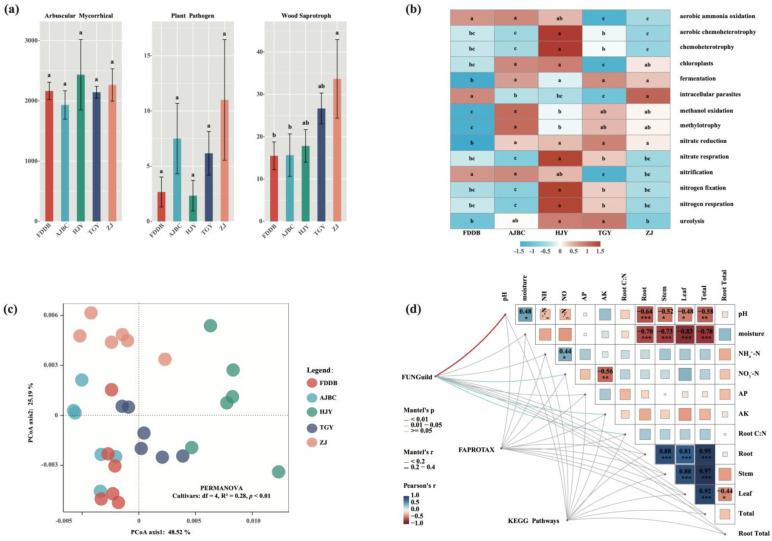
(**a**) Rhizosphere fungal function profiles of different tea cultivars, different letters indicate significant differences between treatments, *p* < 0.05; (**b**) Heatmap results of FAPROTAX function prediction under the rhizosphere of different tea cultivars, the different letters in each row of the figure indicate significant differences between the treatments, *p* < 0.05; (**c**) PCoA of the rhizosphere bacterial KEGG pathways under different tea cultivars; (**d**) Mantel correlation between rhizosphere microbial function profiles and environmental factors under different tea cultivars, Root:Total represents the ratio of Root biomass to total biomass, and Root C:N represents the carbon-nitrogen ratio of roots. *, **, and *** indicate significance levels of *p* < 0.05, *p* < 0.01, and *p* < 0.001, respectively.

FUNGuild prediction results indicated that the functional composition of fungal communities in the rhizosphere of different tea cultivars was predominantly comprised of arbuscular mycorrhizal fungi (AMF), with the average number of identifiable AMF sequences exceeding 2000, although no significant differences were observed among different cultivars (Figure 4a). While wood-saprotrophic fungi showed some variation among different tea cultivar rhizospheres, their overall relative abundance was negligible compared to AMF. The rhizosphere of ZJ exhibited the highest number of sequences identified as wood-saprotrophic fungi, yet the average sequence count remained below 40.

FAPROTAX analysis revealed distinct differences in element cycling-related functions of rhizosphere soil bacterial communities among different tea cultivars (Figure 4b). Notably, bacterial communities in HJY rhizosphere displayed overall enhanced nitrogen cycling-related functions compared to other cultivars. In contrast, bacterial community functions in FDDB rhizosphere were generally weaker than in other cultivars, except for aerobic ammonia oxidation and nitrification, two nitrogen cycling-related functions that showed enhancement. In addition, the KEGG metabolic pathways in the rhizosphere bacterial communities of different tea plant varieties also showed significant differences (Cultivars: df = 4, R^2^ = 0.28, *p* < 0.01) (Figure 4c).

Mantel correlation analysis demonstrated that no significant correlations existed between the functions of bacterial communities in different tea cultivar rhizospheres and either rhizosphere soil physicochemical factors or tea plant physiological characteristics (*p* > 0.05) (Figure 4d). However, the functional composition of fungal communities in different tea cultivar rhizospheres showed significant correlations with rhizosphere soil pH (*p* < 0.01), NO_3_^−^-N (*p* < 0.05), and available potassium (AK) (*p* < 0.05). Additionally, the root carbon-to-nitrogen ratio of tea plants was also significantly correlated with rhizosphere fungal community functions (*p* < 0.05).

## 3. Discussion

During the ongoing domestication by humans, tea plants have gradually diverged into two distinct groups: large-leaf cultivars and small- to medium-leaf cultivars. Concurrently, different tea cultivars have also exhibited regional differentiation. For example, due to differences in cold resistance, large-leaf tea cultivars are predominantly distributed in southern regions, while small- to medium-leaf cultivars have a broader distribution spanning both northern and southern areas [5,6]. Consequently, the physiological characteristics of different tea cultivars have undergone various changes during long-term domestication. In this study, we investigated the diversity, composition, structure, and assembly processes of rhizosphere microbial communities across five tea cultivars widely cultivated in China to elucidate how different tea cultivars influence their rhizosphere microbiomes.

Our results revealed distinct differentiation in physiological characteristics among FDDB, AJBC, HJY, TGY, and ZJ. For instance, FDDB exhibited a clear advantage in biomass compared to other cultivars, while HJY showed no such advantage in biomass. However, the ratio of root biomass to total biomass remained consistent across different tea cultivars without significant variation (Table 1). The predominance of organic acids, fatty acids, and carbohydrates in tea rhizospheres serves critical ecological functions beyond simple carbon provision. Organic acids (e.g., palmitic acid, neotrehalose) chelate nutrients like phosphorus and iron, making them bioavailable while simultaneously acidifying the rhizosphere to favor acidophilic microbes. Fatty acids function as antimicrobial agents, selectively inhibiting pathogens while promoting beneficial taxa. Carbohydrates fuel rapid copiotrophic bacterial proliferation, explaining the observed high bacterial diversity. These cultivar-specific metabolite profiles essentially act as chemical filters, determining which microbial guilds can colonize and persist in each cultivar’s rhizosphere. The distinct metabolite compositions among cultivars (Table S1 and Figure S1) may be attributed to variations in root exudates among cultivars. The five cultivars selected in this study exhibited pronounced differences in physiological characteristics. For example, AJBC, HJY, and ZJ are albino, etiolated, and purple-leaf cultivars, respectively, with significant differences in chlorophyll content that lead to variations in photosynthetic efficiency and products among different cultivars [6]. Furthermore, differences in nutrient-use efficiency among cultivars represent another major factor contributing to variation in rhizosphere soil physicochemical properties [17].

Previous studies have shown that the alpha diversity of bacterial communities in tea rhizosphere soil is generally low. However, the bacterial Chao1 indices in the rhizosphere soil of different tea cultivars in the present study were significantly higher than previously reported [18,19,20], with an average value of approximately 16,000. This elevated diversity can be explained by three interrelated ecological mechanisms. First, the minimal fertilizer application used to maintain normal tea plant growth in our pot experiment reduced chemical stress, allowing stress-sensitive bacterial taxa to persist. Chemical fertilizer application and frequent tillage in conventional field systems create environmental filtering that eliminates oligotrophic and stress-sensitive bacterial groups, thereby reducing overall diversity [10]. Second, the absence of tillage disturbance in pots allowed undisturbed microhabitat development, promoting spatial niche differentiation and enabling coexistence of functionally redundant taxa. Third, the abundance of organic acids, carbohydrates, and other labile metabolites in tea rhizospheres (Table S1) creates resource-rich conditions that support copiotrophic bacterial proliferation. This is evidenced by the dominance of Proteobacteria (~25%), Acidobacteria (~18%), and Actinobacteria (~15%) (Figure 1c)—phyla characteristic of nutrient-enriched, high-fertility soils [21]. The positive relationship between metabolite diversity and bacterial richness suggests that cultivar-specific metabolite portfolios expand the available niche space, allowing more bacterial taxa to coexist through resource partitioning.

The diversity of fungal communities in the rhizosphere of different tea cultivars declined markedly compared to bacterial communities. Compared with most previous studies, both diversity and richness were notably lower, although a few studies have reported similar results. The overall fungal community composition did not differ substantially from previous research, with Ascomycota, Basidiomycota, and Mortierellomycota remaining the dominant phyla in the fungal community structure [8]. Notably, however, Glomeromycota accounted for approximately 11% of the total relative abundance of rhizosphere fungal communities in this study, a result markedly different from previous findings. They are rarely detected in production tea plantations due to fertilization and frequent tillage, with only a few studies reporting high AMF abundance in ancient tea gardens [22]. In the present study, the relative abundance of AMF in the rhizosphere of potted tea plants was considerably higher than in field tea plantation soils, suggesting that tea rhizospheres can enrich AMF. In contrast, fertilization and other agronomic practices lead to a sharp decline in AMF abundance in tea plantation soils. This finding aligns with previous research indicating that fertilization or frequent tillage reduces AMF colonization in plant root systems [23,24].

Furthermore, the pH of tea plantation rhizosphere soil is generally low. While these studies suggest that lower pH is negatively correlated with AMF abundance, prolonged low pH conditions may also induce strong selection effects, allowing only pH-tolerant AMF to survive. Therefore, soil pH may not be the primary cause of AMF decline. Recent research has shown that AMF communities are sensitive to other soil environmental factors rather than directly to soil pH, with soil nutrient availability having a more significant impact on AMF abundance and diversity than pH [22,25]. Taken together, we speculate that appropriately reducing nutrient inputs in tea plantations may promote the recovery of soil AMF, thereby contributing to the healthy and sustainable development of the tea industry.

Previous studies have demonstrated that variations in soil physicochemical properties are the primary drivers of differences in tea rhizosphere microbial communities, particularly soil pH and active aluminum content, though these changes are mainly caused by external fertilization [26]. While our study investigated variations in microbial community structure among different tea cultivars (Figure 2). Rhizosphere soil physicochemical factors explained a substantial proportion of the variation in both bacterial and fungal community structures, consistent with previous research findings [27,28]. Furthermore, this study conducted an in-depth analysis of how rhizosphere metabolites and tea plant physiological factors contribute to variations in rhizosphere bacterial and fungal community structures. The results showed that rhizosphere metabolites explained bacterial and fungal community differences second only to soil physicochemical factors, whereas tea plant physiological factors contributed less to microbial variation. Given that untargeted rhizosphere metabolomics captures both root-derived and microbially produced metabolites, the strong explanatory power of metabolite profiles, particularly for fungal communities, likely reflects combined plant–microbe and microbe–microbe interactions [29]. Bacterial metabolites can influence slower-growing fungal taxa, suggesting that tea rhizosphere fungal communities may be more susceptible to the broader rhizosphere chemical environment than bacterial communities.

The assembly of rhizosphere microbial communities is a complex process influenced by multiple factors. Numerous studies have shown that rhizosphere microbial community composition is regulated by both biotic and abiotic factors, with soil exerting profound effects on the composition of rhizosphere bacterial and mycorrhizal fungal communities [30,31,32,33]. For instance, the complex physicochemical properties of soil can influence plant physiology and root exudation patterns, thereby affecting rhizosphere microbial community composition. Moreover, a study of rhizosphere bacterial microbiomes across different *Arabidopsis* ecotypes demonstrated that soil type strongly influences microbial community assembly [34,35]. In contrast, another study found that rhizosphere actinobacterial communities of strawberry plants grown in different soils were more similar to each other than the bulk soil communities across soils, suggesting that, in this case, the plant is a stronger determinant of microbial community composition than soil type [36]. These findings are consistent with our results, as both null model-based MST and NCM analyses indicated that deterministic processes dominated the assembly of bacterial and fungal communities in the tea rhizosphere. However, while deterministic processes dominated across all cultivars (Figure 3), HJY exhibited distinctive patterns, the lowest bacterial diversity yet high fungal diversity indicated that HJY had higher tolerance of fungal species to acidic environments than bacterial species, which may further explain why HJY deviated from these patterns in fungal assembly. Furthermore, RDA results provided supporting evidence that the influence of tea plants themselves (rhizosphere metabolites and physiological indicators) on rhizosphere microbial community composition exceeded that of soil physicochemical factors. Procrustes analysis further demonstrated the close association between tea rhizosphere metabolites and rhizosphere microbial community assembly. Additionally, studies on crops such as rice, maize, potato, and cucumber have found that different plant cultivars can significantly affect their rhizosphere microbiomes [33,37,38,39]. These findings align with our results, showing that rhizosphere microbial communities differ among tea cultivars, indicating that different tea cultivars significantly impact the assembly of their rhizosphere microbial communities. Furthermore, a field experiment reported that approximately 4–9% of species in the rhizosphere microbial communities were dependent on potato cultivar when three potato varieties were grown at two distant farms [33]. Other studies comparing differences in rhizosphere microbial diversity among inbred maize lines have provided insights into the effects of plant genetics on rhizosphere microbial community composition [39,40]. These findings suggest that by exploiting genetic variation within host plant species, it may be possible to integrate rhizosphere microbial communities into plant breeding programs to promote plant–microbe interactions.

### Practical Applications and Study Limitations

Our findings provide a foundation for cultivar-specific rhizosphere management. For example, carbon and nitrogen cycling functions were relatively enriched in the rhizosphere of AJBC, HJY, and TGY, indicating that more organic fertilizers could be applied to substitute chemical fertilizers. Moreover, high-yield TGY, dominated by saprotrophic *Gymnopilus* (38%), would benefit from autumn organic matter incorporation and delayed spring fertilization. All cultivars require conservation tillage to preserve elevated AMF abundance (11%).

However, several limitations warrant consideration. First, our pot experiment constrained root growth and altered microbial dispersal compared to field systems, potentially amplifying cultivar effects through concentrated root exudation in limited soil volumes. Absolute diversity values and spatial heterogeneity may differ under open-field conditions. Second, while strong correlations exist between cultivar genotypes, metabolites, and microbial communities, our observational design cannot establish definitive directional causality. The cultivar–metabolite–microbiome pathway is inference-based and experimental manipulation (metabolite amendments, reciprocal transplants) would be required to demonstrate causation. Third, our functional predictions using FAPROTAX and FUNGuild are taxonomic inferences, not direct activity measurements. These tools rely on incomplete reference databases that may not capture functional plasticity in tea rhizospheres, and many taxa remain uncharacterized. Predicted functions require validation through metatranscriptomics or enzyme assays. Despite these limitations, our study demonstrates that tea cultivar genotypes significantly shape rhizosphere microbiome assembly through metabolic differentiation, providing a framework for cultivar-specific microbiome management in sustainable tea production.

## 4. Materials and Methods

### 4.1. Experimental Design

The pot experiment was conducted in an open greenhouse covered by sunshade net (which can be called semi-field conditions) at Shengzhou Station, which is affiliated with the Tea Research Institute, Chinese Academy of Agricultural Sciences, located in Shengzhou, Shaoxing City, Zhejiang Province, China (29°74′ N, 120°82′ E). This region is characterized by a typical subtropical climate, with a mean annual temperature of 12.6 °C, a mean annual precipitation of 1200 mm, and an altitude of 23 m. The experimental soil texture revealed a particle size distribution of 1.49% sand, 30.00% silt, and 68.51% clay. The fundamental physicochemical properties of the soil are detailed in Table 3.

Five tea cultivars widely cultivated across China were selected for this study, including *C. sinensis* (L.) O.Kuntze cv. Baiye 1 (AJBC), *C. sinensis* (L.) O.Kuntze cv. Fuding Dabaicha (FDDB), *C. sinensis* (L.) O.Kuntze cv. Huangjinya (HJY), *C. sinensis* (L.) O.Kuntze cv. Tie guanyin (TGY), and *C. sinensis var. assamica* (Masters) Kitamura cv. Zijuan (ZJ). The tea seedlings (cuttings) were transplanted in 2017 and harvested in late October 2018. To ensure experimental homogeneity, seedlings exhibiting uniform growth were selected. Each pot contains 20 kg of soil. The study employed a completely randomized design with six replicates for each cultivar. Agronomic management was performed according to standard practices; specifically, approximately 5 g of a tea-specific compound fertilizer (N-P-K ratio: 18-8-12) was applied per pot annually to ensure plant growth.

### 4.2. Sample Collection and Measurements

Whole tea plants were carefully removed from the pots to preserve root integrity. The rhizosphere soil, defined as the soil layer tightly adhering to the root surface (approximately 1 mm thickness), was separated from the bulk soil. This rhizosphere soil was carefully collected by brushing, placed in sterile tubes, and transported to the laboratory on ice for subsequent analysis. A total of 30 independent samples were collected, with six replicates per tea cultivar. All the samples were processed individually for the following measurements.

Following soil collection, the tea plants were fractionated into three parts, including roots, stems, and leaves. These plant tissues were thoroughly washed with deionized water to remove any residual soil particles and blotted dry with absorbent paper. The samples were then placed in paper envelopes and oven-dried at 60°C to a constant weight to determine the dry biomass of each tissue type. Soil pH was measured using an ORION 3 STAR pH meter (Thermo Scientific, Waltham, MA, USA) at a soil-to-water ratio of 1:2.5 (*w*/*v*). Soil NH_4_^+^-N and NO_3_^−^-N were extracted with 2 M KCl solution (1:10 soil-to-solution ratio) and quantified using a SKALAR SAN++ continuous flow analyzer (Skalar Analytical, Breda, The Netherlands). Available phosphorus (P) and potassium (K) were extracted using the Mehlich-3 universal extraction method and determined via Inductively Coupled Plasma Atomic Emission Spectroscopy (ICP-AES). For plant nutrient analysis, the dried root samples were ground to a fine powder using a pulverizer. Total carbon (C) and nitrogen (N) contents of the root tissues were then determined using an elemental analyzer (CN analyzer).

### 4.3. Microbiome Sequencing and Rhizosphere Soil Metabolomics

Rhizosphere soil total DNA was extracted using the PowerSoil kit (MOBIO Laboratories, Carlsbad, CA, USA) according to the manufacturer’s protocol. The V4–V5 regions of the bacterial 16S rRNA genes were amplified using primers 515F (5′–GTGCCAGCMGCCGCGG–3′) and 907R (5′–CCGTCAATTCMTTTRAGTTT–3′). The internal transcribed spacer 2 (ITS2) regions of the fungal ribosomal RNA gene were amplified using primers ITS3F (5′–GCATCGATGAAGAACGCAGC–3′) and ITS4R (5′–TCCTCCGCTTATTGATATGC–3′). The PCR reaction system and conditions were adapted from our previous research [41]. The resulting PCR amplicons were purified using the QIAquick Gel Extraction Kit (Qiagen, CA, USA) following the manufacturer’s instructions. The purified PCR products were sequenced using the high-throughput sequencing platform (MiSeq PE250, Illumina, San Diego, CA, USA). The microbial sequence data will be available in NCBI by the accession number: PRJNA1401540

Rhizosphere soil samples were freeze-dried prior to metabolite extraction. A 100 mg of sample was weighted to an EP tube after freeze-drying, and 1000 μL extract solution (acetonitrile:methanol:water = 2:2:1, with isotopically labelled internal standard mixture) was added. After 30 s vortex, the samples were homogenized at 35 Hz for 4 min and sonicated for 5 min on ice. The homogenization and sonication cycle was repeated for 3 times. Then the samples were incubated for 1 h at −40 °C and centrifuged at 12,000 rpm for 15 min at 4 °C. The resulting supernatant was transferred to a fresh glass vial for analysis. The quality control (QC) sample was prepared by mixing an equal aliquot of the supernatants from all of the samples. LC-MS/MS analyses were performed using an UHPLC system (Vanquish, Thermo Fisher Scientific, Waltham, MA, USA) with a UPLC BEH Amide column (2.1 mm × 100 mm, 1.7 μm) coupled to Q Exactive HFX mass spectrometer (Orbitrap MS, Thermo, Waltham, MA, USA). The mobile phase consisted of 25 mmol/L ammonium acetate and 25 ammonia hydroxide in water (pH = 9.75) (A) and acetonitrile (B). The analysis was carried with elution gradient as follows: 0~0.5 min, 95%B; 0.5~7.0 min, 95~65% B; 7.0~8.0 min, 65~40% B; 8.0~9.0 min, 40% B; 9.0~9.1 min, 40~95% B; 9.1~12.0 min, 95% B. The column temperature was 25 °C. The auto-sampler temperature was 4 °C, and the injection volume was 3 μL. The QE HFX mass spectrometer was used for its ability to acquire MS/MS spectra on information-dependent acquisition (IDA) mode in the control of the acquisition software (Xcalibur version 1.8, Thermo). In this mode, the acquisition software continuously evaluates the full scan MS spectrum. The ESI source conditions were set as following: sheath gas flow rate as 50 Arb, Aux gas flow rate as 10 Arb, capillary temperature 320 °C, full MS resolution as 60,000, MS/MS resolution as 7500, collision energy as 10/30/60 in NCE mode, spray Voltage as 3.5 kV (positive) or −3.2 kV (negative), respectively.

The raw data were converted to the mzXML format using ProteoWizard (version 3.0) and processed with an in-house program developed in R and based on XCMS for peak detection, extraction, alignment, and integration. Then an in-house MS2 database (BiotreeDB) was applied in metabolite annotation (Shanghai Biotree Biotech Co., Ltd., Shanghai, China). The annotation cutoff was set to 0.3. The details of quality control are also visualized in Figure S3, scores of PCA plot, and the correlations analysis revealed the high data quality of metabolomics analysis.

### 4.4. Bioinformatics and Statistical Analysis

Raw sequencing data were processed using USEARCH (v11.0.667) [42]. Initially, paired-end reads were merged, and the resulting sequences were corrected against the RDP database [43] for bacteria and the UNITE database [44] for fungi. Basic sequence statistics, including minimum, maximum, and average lengths, were assessed using the *fastx-utils* command. Subsequently, sequences were quality-filtered using *fastq_filter* to discard reads shorter than the average sequence length or those exceeding an expected error threshold of 0.4. Unique sequences were identified using the *fastx_uniques* command. Denoising and chimera removal were performed using the unoise3 algorithm to generate a ZOTU table using *otutab*. Taxonomic classification was conducted using *sintax*, assigning bacterial sequences against the RDP database at a 97% similarity threshold and fungal sequences against the UNITE database at an 80% similarity threshold. Finally, the minimum sequence count per sample was determined using the *min_size* command in atlas-utils, and the ZOTU table was rarefied using *otutab_rare* to standardize sequencing depth for downstream analyses.

All statistical analyses were performed using R software (version 4.2.2). Alpha diversity indices (Chao1 and Shannon) were calculated using the USEARCH *alpha_div* command, and differences between treatments were evaluated using the Kruskal–Wallis test. Microbial community composition was characterized by the relative abundance of the top ten phyla. Significant differences in composition were assessed using one-way Analysis of Variance (ANOVA) followed by the Least Significant Difference (LSD) test. Bacterial and fungal community structures were visualized at both the phylum and genus levels. Beta diversity was evaluated via Principal Coordinates Analysis (PCoA) based on Bray–Curtis distances. The significance of community structural differences among treatments was tested using Permutational Multivariate Analysis of Variance (PERMANOVA) with the vegan package. Redundancy Analysis (RDA) was employed to assess the contribution of rhizosphere metabolites, soil factors, and plant factors to variations in microbial community structure. Additionally, Random Forest (RF) models were applied to predict the specific contributions of these factors to bacterial and fungal community dynamics (packages: randomForest, rfPermute, and rfUtilities).

The relative importance of deterministic versus stochastic processes in microbial community assembly was evaluated using the Neutral Community Model (NCM) and the Modified Stochasticity Ratio (MST) [45]. An MST value of 0.5 served as the threshold boundary: MST < 0.5 indicated a deterministic-dominated assembly, while MST > 0.5 indicated a stochastic-dominated assembly. Calculations were performed using the NST package based on Bray–Curtis and Jaccard distances (excluding phylogenetic diversity).

Bacterial functional profiles were predicted using PICRUSt2 [46]. The annotated ZOTU table and representative sequences were processed via the picrust2_pipeline.py script, with functional descriptions added using add_descriptions.py. Alternatively, bacterial functions were predicted using FAPROTAX by processing the annotated ZOTU table with the collapse_table.py script [47]. Fungal functional guilds were predicted using FUNGuild via the Guilds_v1.1.py script applied to the annotated fungal ZOTU table [48].

Rhizosphere metabolomic data were normalized, and metabolites with a secondary match score (MS2 score) > 0.9 were retained to ensure high identification confidence. Partial Least Squares Discriminant Analysis (PLS-DA) was performed using the ropls package in R. The dimensionality-reduced data from the PLS-DA axes were retained to represent the rhizosphere metabolome in combined analyses with microbial data. Variable Importance in Projection (VIP) scores were calculated using the getVipVn function; metabolites with VIP > 1 were selected to test for significant differences between varieties using the LSD test (*p* < 0.05).

To evaluate the concordance between rhizosphere metabolites and microbial communities, Procrustes Analysis (PA) was conducted using the phyloseq package. Finally, differences in soil and plant factors among tea varieties were analyzed using the LSD test.

## 5. Conclusions

This study establishes that tea cultivar genotype could be a critical determinant of rhizosphere microbiome assembly, operating through cultivar-specific metabolic signatures that selectively shape microbial communities. Our findings indicate that rhizosphere metabolite profiles may serve as key mediators linking plant genotype to microbial community structure, with deterministic processes rather than stochastic factors governing microbiome assembly across cultivars. The strong coupling between rhizosphere metabolomes and microbial communities underscores the importance of plant-microbe chemical communication in structuring below-ground ecosystems. Also, these findings have significant implications for tea cultivation practices and breeding programs. Integrating rhizosphere microbiome considerations into cultivar selection and developing cultivar-specific management strategies could enhance plant–microbe interactions, thereby improving plantation sustainability, reducing dependence on chemical inputs, and promoting ecosystem health. Future research should validate these findings from controlled environments under field conditions and explore the functional consequences of cultivar-driven microbiome differences for tea plant performance, soil health, and product quality.

## Figures and Tables

**Table 1:** Rhizosphere soil properties and plant indices differences among different tea cultivars (mean ± se, n = 6).

Factor	Tea Cultivars				
	AJBC	FDDB	HJY	TGY	ZJ
pH	3.63 ± 0.01 b	3.6 ± 0.02 b	3.72 ± 0.01 a	3.7 ± 0.02 a	3.69 ± 0.01 a
NH_4_^+^-N mg kg^−1^	24.43 ± 3.39 a	22.73 ± 3.37 ab	15.11 ± 1.28 b	24.3 ± 3.32 a	29.24 ± 0.39 a
NO_3_^−^-N mg kg^−1^	4.82 ± 0.5 b	5.66 ± 0.62 b	3.9 ± 0.26 b	7.77 ± 0.88 a	4.22 ± 0.74 b
AK mg kg^−1^	26.63 ± 0.64 c	26.94 ± 0.88 bc	29.3 ± 0.36 ab	26.27 ± 1.02 c	30.19 ± 1.15 a
AP mg kg^−1^	2.15 ± 0.67 ab	1.16 ± 0.19 b	1.11 ± 0.19 b	1.15 ± 0.36 b	2.94 ± 0.79 a
Moisture %	0.34 ± 0 bc	0.28 ± 0.01 d	0.37 ± 0.01 a	0.31 ± 0.01 cd	0.34 ± 0.01 ab
Root biomass g	28.94 ± 2 b	44.45 ± 1.19 a	17.48 ± 1.61 c	28.33 ± 2.21 b	27.43 ± 2.21 b
Stem biomass g	26.29 ± 3.13 b	43.02 ± 3.27 a	15.9 ± 2.49 c	25.76 ± 1.88 b	25.74 ± 2.26 b
Leaf biomass g	16.83 ± 1 b	23.46 ± 1.74 a	8.6 ± 0.66 d	18.57 ± 0.93 b	13.1 ± 0.93 c
Total biomass g	72.06 ± 5.08 b	110.93 ± 5.65 a	41.97 ± 4.61 c	72.65 ± 4.26 b	66.26 ± 4.47 b
Root:Total	0.41 ± 0.02 a	0.4 ± 0.01 a	0.42 ± 0.01 a	0.39 ± 0.02 a	0.41 ± 0.02 a
Root C:N	56.4 ± 2.15 a	58.66 ± 1.71 a	50.28 ± 3.02 a	55.79 ± 6.64 a	49.08 ± 2.47 a

Note: Root:Total represents the ratio of Root biomass to total biomass, and Root C:N represents the carbon-nitrogen ratio of roots. In the table, different letters in the same row indicate significant differences among different varieties, with *p* < 0.05.

**Table 2:** Variance decomposition results of rhizosphere bacterial and fungal communities RDA from different tea cultivars.

Factors	Bacterial Community	Fungal Community
Soil properties explained	23.40%	24.93%
Plant indices explained	13.41%	11.88%
Metabolism explained	19.87%	21.62%
Total explained	64.75%	70.82%

**Table 3:** Basic soil properties of the pot experiment.

Soil Properties	Values
pH	4.46
TN g kg^−1^	1.24
SOC g kg^−1^	10.08
AP mg kg^−1^	1.84
AK mg kg^−1^	103.85

TN means total nitrogen; SOC means soil organic carbon; AP means available phosphorus; AK means available potassium.

## Data Availability

Data will be made available on request.

## Supplementary Materials

The following supporting information can be downloaded at: https://www.mdpi.com/article/10.3390/plants15030414/s1, Table S1. The metabolism differences among different tea cultivars; Figure S1. PLS-DA for the rhizosphere metabolome of different tea cultivars; Figure S2. Neutral community model of the rhizosphere bacterial and fungal communities in different tea cultivars. R^2^ represents the goodness of fit of the model and m represents the migration rate of the species in the community. A, B, C, D and E are the neutral community models for rhizosphere bacteria, and F, G, H, I and J are the neutral community models for rhizosphere fungi, respectively. Figure S3. Positive (a) and negative (b) ion PCA score plot. The green dots are QC samples, and the blue dots are formal experimental samples. Correlation analysis between QC samples and positive (c) and negative (d) ions, respectively.
