# Supplementary material for: Tea Cultivar Genotype Shapes Rhizosphere Microbiome Assembly Through Metabolic Differentiation
Source: Plants (Basel). 2026 Jan 29;15(3):414. doi: 10.3390/plants15030414 (PMC12899138; doi:10.3390/plants15030414)
Supplement: Supplementary file 1 [file plants-15-00414-s001.zip › Supplementary Table.pdf]

Table S1 The metabolism differences among different tea cultivars

MS names	AJBC	FDDB	HJY	TGY	ZJ
Eicosadienoic acid	0.05±0.01b	0.26±0.06a	0.08±0.01b	0.11±0.02b	0.07±0.01b
Pentadecanoic acid	1.87±0.19ab	1.77±0.12b	1.66±0.09b	1.59±0.15b	2.19±0.12a
Tridecanoic acid	0.25±0.05a	0.14±0b	0.17±0.04ab	0.14±0.01b	0.17±0.03ab
4-Dodecylbenzenesulfonic Acid	0.67±0.02ab	0.67±0.03ab	0.67±0.02ab	0.62±0.02b	0.7±0.03a
15-Methylpalmitate	0.94±0.1c	1.5±0.11a	1.19±0.06bc	0.98±0.08bc	1.21±0.09b
Tetracosanoic acid	0.52±0.06b	0.51±0.04bc	0.33±0.08c	0.72±0.08a	0.45±0.04bc
Maslinic acid	0.34±0.07a	0.32±0.05a	0.46±0.07a	0.47±0.18a	0.22±0.03a
Palmitic acid	25.71±1.98c	38.21±1.58a	32.27±1.23b	29.72±2.56bc	29.01±1.72bc
Dodecanoic acid	0.75±0.08b	2.2±0.23a	1.46±0.11ab	1.02±0.11b	2.19±0.51a
Tiglic acid	0.06±0.01c	0.12±0.01ab	0.14±0.02a	0.09±0.01bc	0.07±0.01c
Taurine	0.67±0.07a	0.67±0.06a	0.59±0.01ab	0.54±0.01b	0.56±0.02ab
Theophylline	0.09±0.05bc	0.02±0.01c	0.58±0.11a	0.5±0.32ab	0.07±0.07bc
Mannitol	1.76±0.67bc	0.31±0.07c	4.04±0.34a	2.25±0.87b	0.82±0.2bc
Ribitol	0.58±0.13a	0.25±0.03b	0.69±0.05a	0.23±0.08b	0.27±0.04b
9,10-DHOME	0.32±0.09c	1.12±0.22a	0.28±0.03c	0.68±0.08b	0.41±0.05bc
5Z-Dodecenoic acid	0.02±0b	0.03±0.01b	0.09±0.02b	0.03±0.02b	0.56±0.2a
Adenine	0.33±0.05b	0.58±0.07a	0.48±0.05a	0.25±0.03b	0.33±0.04b
2-（3,4-dihydroxyphenyl）-3,4-dihydro-2H-1-benzopyran-3,5,7-triol	0.41±0.26a	0.34±0.1a	0.23±0.12a	0.41±0.15a	0.16±0.05a
Capric acid	1.22±0.05ab	1.15±0.07ab	1.05±0.1ab	1.24±0.06a	1.03±0.06b
Sucrose	0.9±0.06b	1.27±0.13a	1.15±0.08a	0.82±0.07b	0.72±0.05b
12-Oxo-2,3-dinor-10,15-phytodienoic acid	0.16±0.04b	0.02±0.01c	0.33±0.03a	0.03±0.01c	0.09±0.03bc
2-Hydroxystearic acid	0.22±0.02d	0.61±0.04a	0.38±0.02b	0.29±0.02cd	0.35±0.03bc
Turanose	13.58±0.51b	19.16±1.83a	17.81±1.51a	11.44±1.35bc	9.77±0.26c

Glycolic acid	0.69±0.12b	2.88±0.3a	0.84±0.05b	0.62±0.08b	0.97±0.11b
（-）-Epigallocatechin 3-cinnamate	1.89±0.14b	2.13±0.2b	3.62±0.42a	1.93±0.17b	2.99±0.34a
Trehalose	1.88±0.36b	3.95±0.57a	1.97±0.2b	3.37±0.37a	2.9±0.24ab
PA（18:3（9Z,12Z,15Z）/16:0）	0.57±0.12b	0.58±0.13b	0.51±0.05b	0.53±0.09b	1.13±0.2a
3-Hydroxy-8'-apo-epsilon-caroten-8'-al	2.14±0.54b	0.93±0.35b	19.49±1.72a	1.11±0.26b	1.48±0.21b
LysoPI（20:0/0:0）	0.32±0.02b	0.28±0.03b	3.25±0.56a	0.21±0.03b	0.23±0.02b
beta-Citraurol	10.11±1.59b	4.59±0.83bc	64.05±4.7a	3.05±0.39c	4.13±0.4bc
4-Hydroxybenzaldehyde	0.86±0.15b	1.19±0.13ab	0.89±0.16b	1.33±0.16a	0.88±0.16b
Colubrinic acid	3.04±1.08b	1.4±0.31b	20.17±1.55a	0.79±0.12b	0.56±0.07b
Neotrehalose	129.48±38.04c	603.89±73.26a	227.16±32.49bc	107.79±23.33c	259.59±31.85b
5-HETE	31.62±6.93a	12.33±3.21b	33.43±6.41a	14.9±2.81b	12.82±1.88b
Ganodermic acid TQ	0.15±0.02b	0.05±0.02b	2.81±0.4a	0.04±0.01b	0.04±0.01b
Glycyrrhetinic acid	6.94±1.77b	3.57±0.42bc	31.12±2.46a	2.69±0.19c	2.59±0.15c
Ceanothic acid	1.15±0.26b	0.72±0.15b	11.99±1.44a	0.46±0.05b	0.4±0.06b
Levan	1.62±0.3b	2.67±0.31a	2.44±0.22ab	2.64±0.44a	2.4±0.25ab
Montecristin	0.42±0.13a	0.72±0.18a	0.65±0.11a	0.74±0.2a	0.35±0.11a
8-Hydroxyhyperforin 8,1-hemiacetal	1.12±0.15b	0.31±0.12b	6.63±0.78a	0.19±0.06b	0.36±0.07b
Colupone	3.27±0.55b	1.61±0.27b	19.17±1.8a	1.33±0.13b	1.13±0.09b
