# Supplementary figures and images for: Tea Cultivar Genotype Shapes Rhizosphere Microbiome Assembly Through Metabolic Differentiation
Source: Plants (Basel). 2026 Jan 29;15(3):414. doi: 10.3390/plants15030414 (PMC12899138; doi:10.3390/plants15030414)

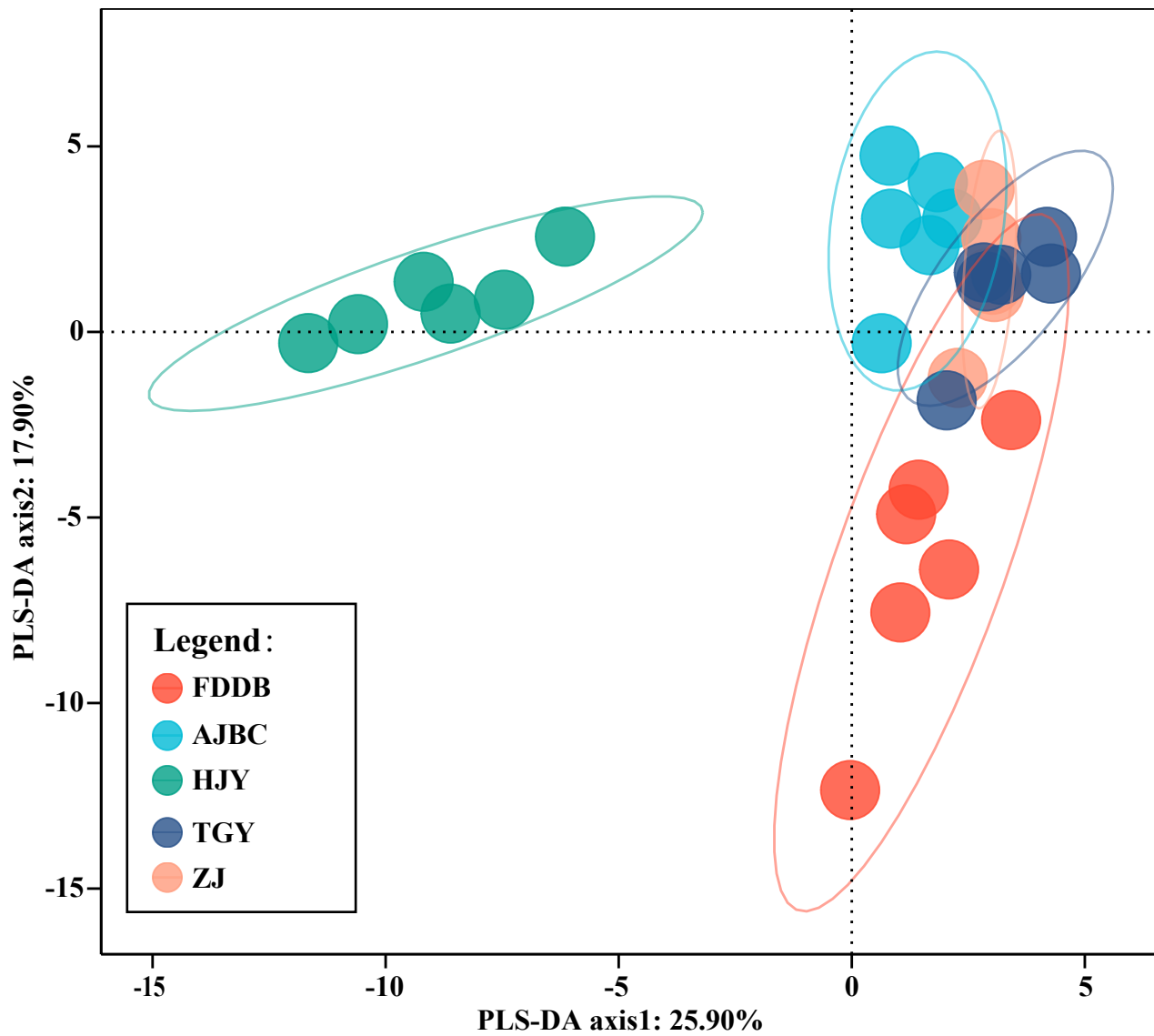

Supplement: Supplementary file 1 [file plants-15-00414-s001.zip › figureS1.pdf]

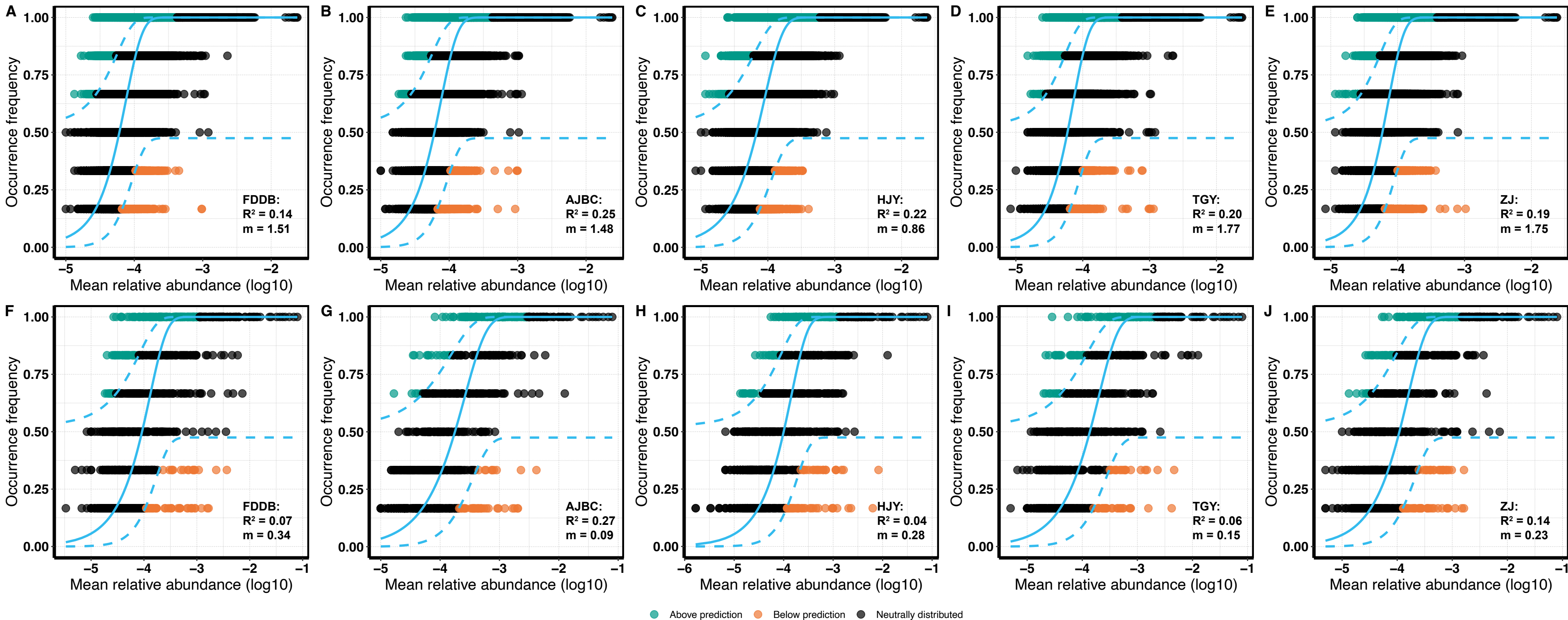

Supplement: Supplementary file 1 [file plants-15-00414-s001.zip › figureS2.pdf]

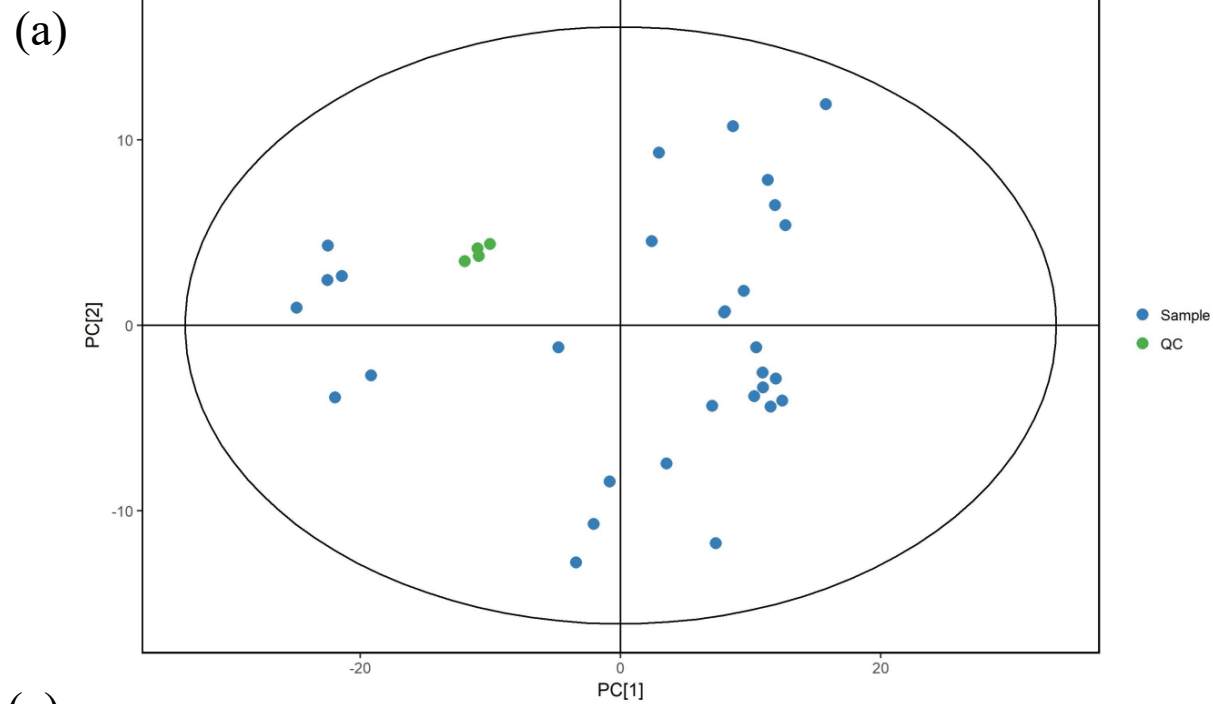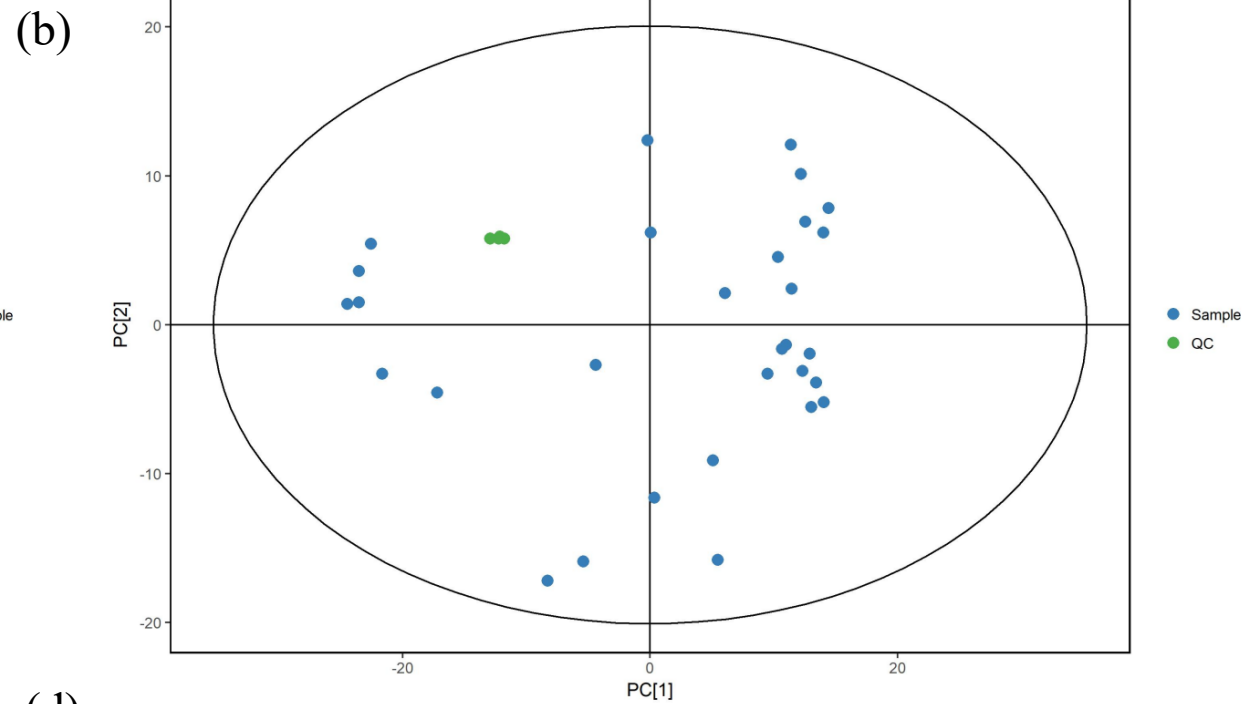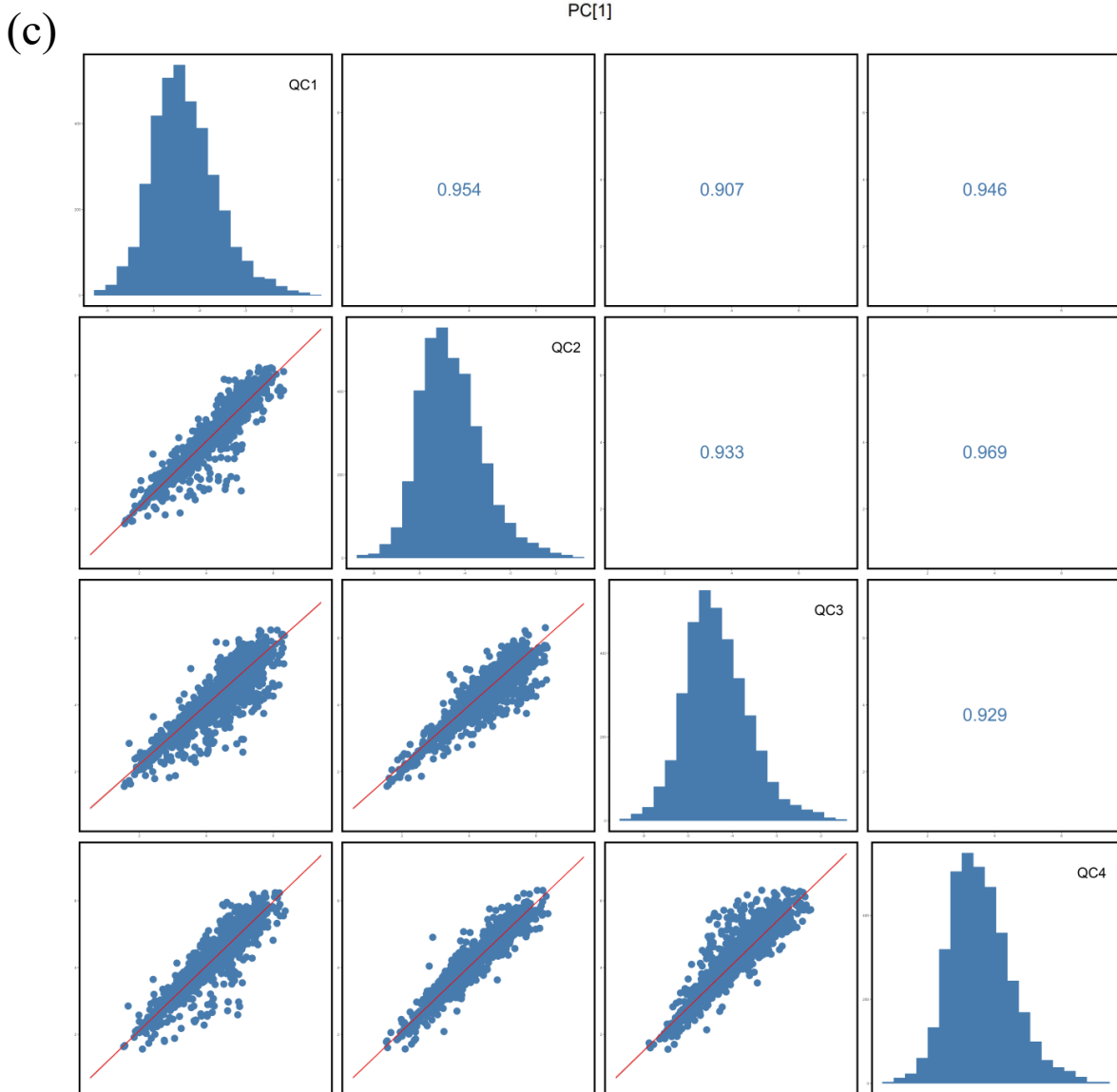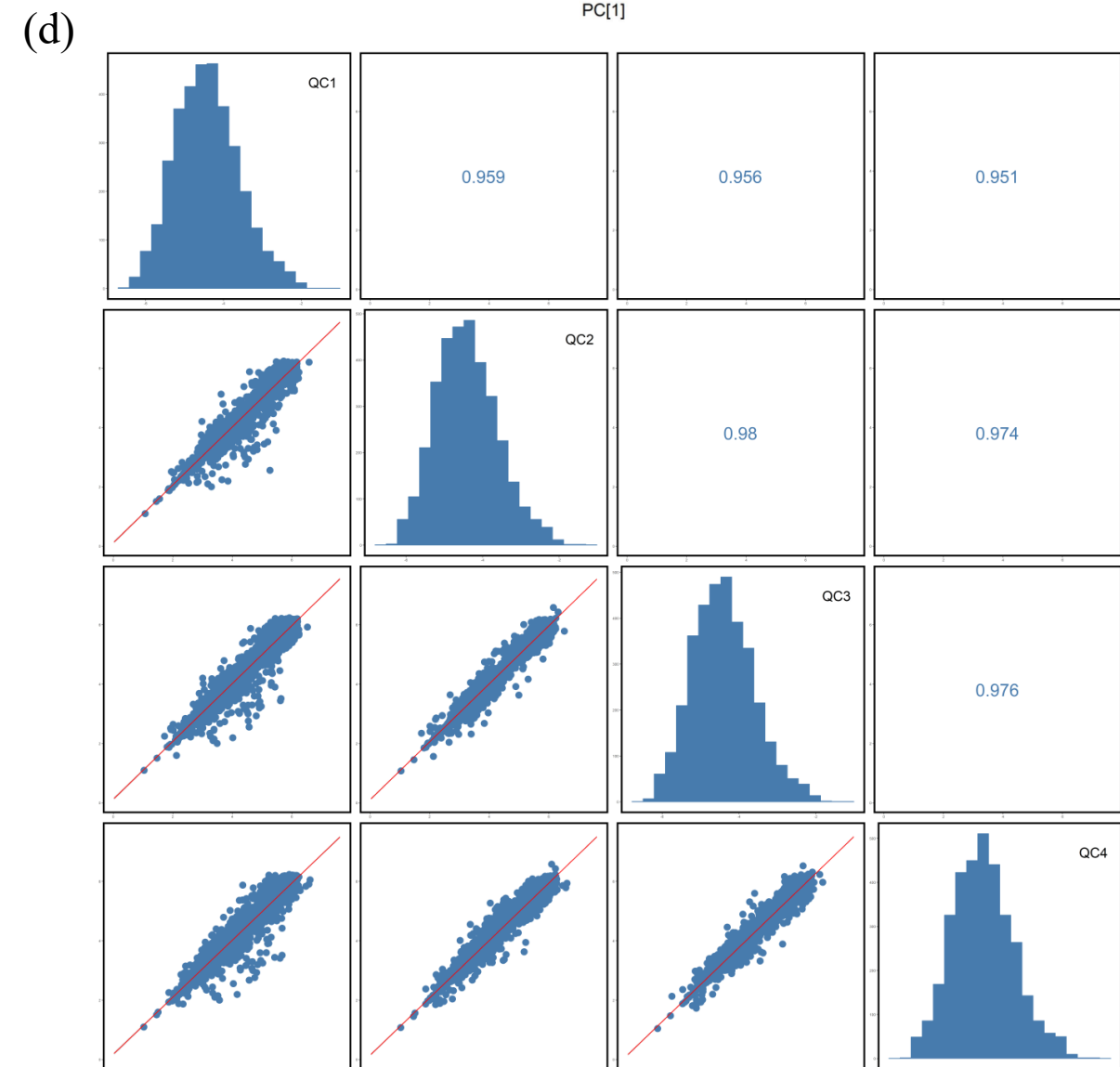

Supplement: Supplementary file 1 [file plants-15-00414-s001.zip › FigureS3.pdf]
